# P011. The use of electronic pain diaries via telemedicine for managing chronic pain

**DOI:** 10.1186/1129-2377-16-S1-A190

**Published:** 2015-09-28

**Authors:** Giovanni Franco, Marianna Delussi, Vittorio Sciruicchio, Walter Marani, Laura De Rocco, Marina de Tommaso

**Affiliations:** Neurophysiopathology of Pain Unit, Neuroscience and Sensory Systems Department, University of Bari “Aldo Moro”, Bari, Italy; Pediatric Neurology Unit, “Giovanni XXIII” Hospital, Bari, Italy; THCS - Consorzio Stabile Terin Parco Tecnologico Cittadella della Ricerca, Mesagne, Brindisi Italy

Chronic pain is defined as pain that persists for longer than 3 to 6 months with persistence beyond “normal healing time” of an injury [1]. Pain is a subjective experience, which is difficult to accurately measure. Current approaches to evaluate chronic pain suffer from methodological problems. A real-time data capture approach using electronic diaries has been proposed as a new standard for pain measurement. The formulation of a correct diagnosis and the delivery of optimal care depend on accurate communication between patients and clinicians regarding patients’ symptoms that necessitate reliance on memory, which is often imprecise. Data suggest that remote clinical assessments via telemedicine can improve clinical monitoring, diagnosis and care, and facilitate research participation.

Our aim was to evaluate the feasibility and the reliability of using a handheld electronic communication device via telemedicine as a method for assessing and monitoring pain and discomfort in chronic pain patients.

In collaboration with TERIN, an Italian ICT (information and communication technology) consortium, we have developed an easy-to-use smartphone-based electronic pain diary (IHCS AID Diary) which enables assessment of clinical features of pain over time. Data are transferred via internet to the central server that provides the web interface to access the system (IHCS - Innovative Health Care System), to which we can connect to explore processed data and to interact with it. Fifty-three headache patients were selected. The subject's task, during pain, was to indicate the location of pain (on a bodymap), the intensity of pain (on a visual analogue scale - VAS), the state of discomfort (on the Wong-Baker FACES pain rating scale), other pain associated manifestations and therapeutic response by using the AID Diary. All subjects also completed paper pain diaries.

Preliminary results showed that 27 patients (51%) were compliant in using the AID Diary during pain, 18 patients (34%) had issues with application malfunctioning and transferring data and 8 patients (15%) were noncompliant. Paper pain diaries returned by the group of compliant patients contained more errors and omissions compared to the AID Diary.

The potential use of a smartphone-based electronic pain diary via telemedicine seems to be a feasible and reliable method for conducting remote assessments of clinical features of pain in chronic pain patients over time, thus improving clinical monitoring, differential diagnosis and treatment of pain.

Written informed consent to publish was obtained from the patient(s).
